# Contrasting Taxonomic and Phylogenetic Diversity Responses to Forest Modifications: Comparisons of Taxa and Successive Plant Life Stages in South African Scarp Forest

**DOI:** 10.1371/journal.pone.0118722

**Published:** 2015-02-26

**Authors:** Ingo Grass, Roland Brandl, Alexandra Botzat, Eike Lena Neuschulz, Nina Farwig

**Affiliations:** 1 Department of Ecology—Conservation Ecology, Faculty of Biology, Philipps-Universität Marburg, Marburg, Germany; 2 Department of Ecology—Animal Ecology, Faculty of Biology, Philipps-Universität Marburg, Marburg, Germany; 3 Department of Ecology, Technische Universität Berlin, Berlin, Germany; 4 Biodiversity and Climate Research Centre (BiK-F) and Senckenberg Gesellschaft für Naturforschung, Frankfurt (Main), Germany; University of Guelph, CANADA

## Abstract

The degradation of natural forests to modified forests threatens subtropical and tropical biodiversity worldwide. Yet, species responses to forest modification vary considerably. Furthermore, effects of forest modification can differ, whether with respect to diversity components (taxonomic or phylogenetic) or to local (α-diversity) and regional (β-diversity) spatial scales. This real-world complexity has so far hampered our understanding of subtropical and tropical biodiversity patterns in human-modified forest landscapes. In a subtropical South African forest landscape, we studied the responses of three successive plant life stages (adult trees, saplings, seedlings) and of birds to five different types of forest modification distinguished by the degree of within-forest disturbance and forest loss. Responses of the two taxa differed markedly. Thus, the taxonomic α-diversity of birds was negatively correlated with the diversity of all plant life stages and, contrary to plant diversity, increased with forest disturbance. Conversely, forest disturbance reduced the phylogenetic α-diversity of all plant life stages but not that of birds. Forest loss neither affected taxonomic nor phylogenetic diversity of any taxon. On the regional scale, taxonomic but not phylogenetic β-diversity of both taxa was well predicted by variation in forest disturbance and forest loss. In contrast to adult trees, the phylogenetic diversity of saplings and seedlings showed signs of contemporary environmental filtering. In conclusion, forest modification in this subtropical landscape strongly shaped both local and regional biodiversity but with contrasting outcomes. Phylogenetic diversity of plants may be more threatened than that of mobile species such as birds. The reduced phylogenetic diversity of saplings and seedlings suggests losses in biodiversity that are not visible in adult trees, potentially indicating time-lags and contemporary shifts in forest regeneration. The different responses of taxonomic and phylogenetic diversity to forest modifications imply that biodiversity conservation in this subtropical landscape requires the preservation of natural and modified forests.

## Introduction

Subtropical and tropical forests are hotspots of global biodiversity [1]. At the same time, they are among the most threatened ecosystems worldwide, with the ongoing intensification of human land-use posing a particularly high risk [2]. Consequently, subtropical and tropical forests are increasingly found only as human-modified forest landscapes [3,4]. This conversion from primary into modified forest has profound effects on the composition of forest communities [4,5]. While, on the one hand, late successional species and forest specialists are highly vulnerable [3,6], on the other, some species persist or even increase in abundance in modified forest landscapes [6–8].

However, the assumption that some species are robust towards forest modification becomes problematic when negative effects are masked by a time-lag between habitat modification and species extinction, resulting in an ‘extinction debt’ [9,10]. Time-lags are especially pronounced in long-lived species [11]. For example, the diversity of adult tree species responds only weakly to forest modification, whereas that of saplings or seedlings can already indicate ongoing changes in the patterns of forest regeneration [12]. Furthermore, negative effects of forest modification are typically more readily visible in mobile species such as birds than in sessile species such as plants [11,13].

To focus on species richness (or, more generally, taxonomic diversity; TD) only ignores important aspects of the overall diversity in ecosystems. Many functional traits of species show a phylogenetic signal, such that phylogenetic diversity (PD) is often a more appropriate indicator of functional processes than TD as well as a potential surrogate for functional diversity in many ecosystems [14]. In fact, a higher PD has been shown to enhance ecosystem functioning and stability [15,16], especially when PD is decoupled from TD [17–19]. In addition, comparisons of observed patterns in PD to models simulating random species’ distributions allow for the study of ecological processes that structure species communities, such as limiting similarity or environmental filtering [20–23].

The relative effects of limiting similarity and environmental filtering on the structure of species communities may vary considerably at the landscape scale. Indeed, human-modified forest landscapes impose strong species-specific effects on the immigration, extinction, and dispersal rates of species, thereby creating a geographic mosaic of biodiversity [24]. The recognition of diversity within a community (α-diversity) and among communities (β-diversity) helps us to understand these processes [19,25]. However, the effects of forest modification on α- and β-diversity, both for TD and for PD, in subtropical and tropical forests are still poorly documented, despite their relevance for conservation management and planning [19]. An informative assessment of α- and β-diversity for both diversity types would benefit from a focus on multiple taxa, as indicators of historic as well as ongoing changes in forest regeneration and community diversity. Tree and bird species are ideally suited for this purpose. For the former, their long generation times and longevity, coupled with their multiple successive life stages, from seedling to adult tree, facilitate direct comparisons of past and contemporary patterns in forest diversity [12]. In fact, previous studies have demonstrated time-lags in the response of tree species to forest loss [11,12]. Birds are well-studied organisms whose local composition strongly reflects within-forest disturbance and local loss in forest cover. Forest-specialist birds in particular are highly dependent on structurally rich and sufficiently large forests [7,26]. Finally, both tree and bird species are vital to a functional forest ecosystem and to its associated services to humans [27,28].

Here we studied the α- and β-diversity within the taxonomic and phylogenetic diversity of trees and birds in a human-modified forest landscape in subtropical South Africa. For trees, we compared three life stages: adult trees, saplings, and seedlings. The study landscape was mainly characterized by highly diverse indigenous scarp forest. Despite being naturally fragmented and often forming small patches, scarp forests are a taxonomically and phylogenetically highly diverse forest type. In South Africa, their conservation is a priority [29,30], as they are increasingly threatened by the intensification and expansion of agriculture as well as by urban sprawl [31]. We selected a wide range of modifications affecting naturally occurring scarp forest that differed in its degree of within-forest disturbance and the size of forest fragments (forest loss). At the outset of the study, it was predicted that increasing forest disturbance and forest loss would: (1) yield contrasting patterns in the TD and PD of species groups; (2) increase the β-diversity of TD and PD, shaping a mosaic of diversity in this human-modified landscape; and (3) negatively affect the α- and β-PD of saplings, seedlings, and birds but impose weaker effects on the α- and β-PD of adult trees than on the other species groups, consistent with a time-lag in the effects of forest modification on forest communities.

## Methods

### Ethics statement

Permission was obtained from Ezemvelo KZN Wildlife to work within protected areas. Private land owners provided permission to work on their land. The following landowners or companies should be contacted for future permits: G. Archibald (Glen Rosa), M.V. Neethling (Minnehaha), and Lake Eland Game Reserve. Field studies did not involve endangered species and no protected species were sampled. All animal work was conducted according to relevant national and international guidelines. Animal studies were observant only; hence no approval by an ethics committee was necessary.

### Study region

This study was conducted in a heterogeneous subtropical landscape situated near the eastern coast of South Africa, within the Vernon Crookes (30°16’S, 30°35’E, 2189 ha) and Oribi Gorge (30°40’S, 30°18’E, 1850 ha) Nature Reserves and in the surrounding agricultural areas. Seasonal mean monthly temperatures ranged from 8 to 28°C and monthly rainfall from 600 to 1200 mm [31]. The natural vegetation of this heterogeneous landscape mainly consists of fragmented patches of indigenous scarp forest interspersed with natural grassland. Scarp forests are at the transition between the South African Afromontane and Indian Ocean belt forests and therefore contain a highly diverse mixture of species from these forest types as well as scarp-forest-specific endemics [29]. Scarp forests often form small, yet highly diverse fragments; their natural fragmentation results from past and contemporary microclimatic and orographic conditions [30]. Due to their large number of endemic species, their biodiversity, and their importance for forest specialists, scarp forests rank high in conservation priority [29,31]; however, the intensive expansion of agricultural land, including monocultures of eucalyptus and sugarcane, together with urban sprawl has, in many cases, led to their degradation. Previous studies in the region found variable effects of the surrounding matrix on the composition of forest communities as well as on ecosystem processes related to forest regeneration [7,32,33]. In this study, to examine the effects of a wide array of forest modifications, we selected the five most widespread types: (1) Large, continuous scarp forests and (2) natural scarp forest fragments surrounded by natural grassland were located within protected areas. Scarp forest fragments in the agricultural matrix were surrounded by either (3) eucalyptus or (4) sugarcane plantations. (5) Secondary forests whose growth followed the severe overgrazing of scarp forests by cattle were also included; these forests mainly consist of *Acacia* species. Both the structural and the size characteristics of the forest fragments were investigated as well (see section “Forest fragment characteristics” and S1 Table).

### Field surveys

**Fig 1:**
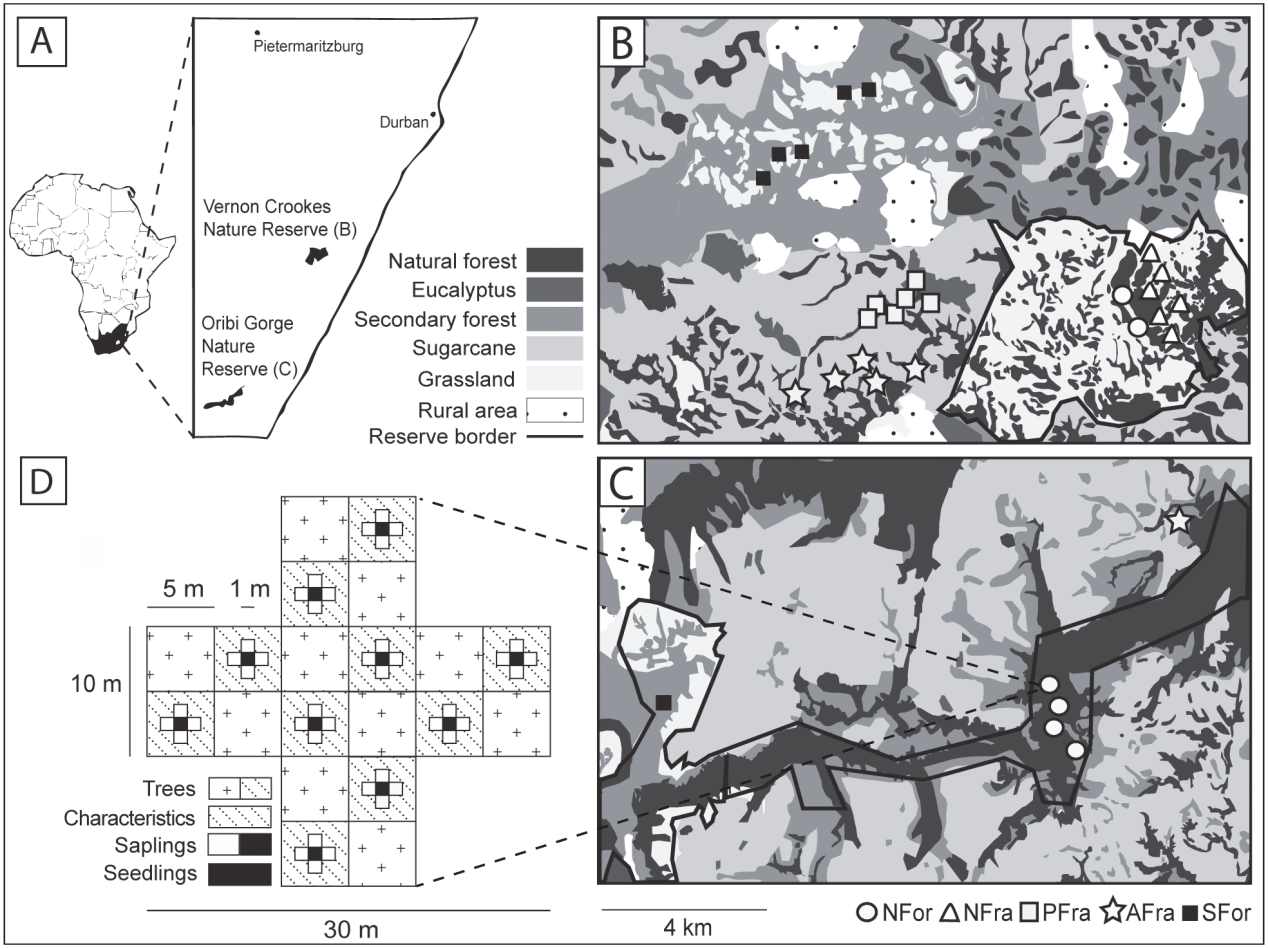
Map of the study area and schematic diagram showing the design of the survey of plant diversity and forest characteristics. (A) The study area in South Africa and the locations of the thirty study plots used for tree surveys within and in the surroundings of the (B) Vernon Crookes and (C) Oribi Gorge Nature Reserves. The main habitat and land-use types are depicted in different gray intensities. Symbols in (B) and (C) refer to the five types of forest modification (NFor = large natural scarp forests; NFra = natural scarp forest fragments; PFra = scarp forest fragments within eucalyptus plantations; AFra = scarp forest fragments within sugarcane plantations; SFor = secondary forests). The schematic diagram in (D) shows the study design used to map adult trees, saplings, and seedlings as well as the environmental characteristics within subplots of the 500-m^2^ study plots. Note that only 27 out of the 30 study plots were investigated for bird diversity, but the null model analysis of the phylogenetic diversity of plants was based on data from all 30 study plots. Map modified after [33].

Fieldwork was carried out from January until April 2010 (adult trees, saplings, and seedlings) and from November 2008 until February 2009 (birds). For the plant and bird surveys, six permanent study plots were established for each of the five above-described forest modifications (Fig. 1). Each plot was situated, wherever possible, in a different forest fragment and consisted of two crossing transects 10 m in width and 30 m in length (covered area 500 m^2^). Pairwise distances between study plots ranged from 180 to 64,530 m (mean ± SD: 21,380 ± 24,530 m).

Local plant diversity was assessed across the three different successive life stages of trees: adults, saplings, and seedlings. We are aware that the following definitions of these stages are rough and may differ in their applicability within and among different tree species. However, at least in this study, they allowed an efficient assessment of the diversity of tree species with respect to past processes (adult trees) as well as contemporary forest regeneration (saplings and seedlings). We defined adult trees as those with a diameter at breast height (dbh) > 5 cm or a height > 4 m and mapped all adult individuals in each of the 500-m^2^ plots. To assess sapling diversity, ten 5-m^2^ subplots within each study plot were established and all individuals with a diameter < 5 cm at their base or a height > 75 cm were mapped. For seedlings, we established ten 1-m^2^ subplots, and mapped all individuals with a diameter at base < 1 cm or a height < 75 cm. Species were identified following [34] and [35]. Nomenclature followed the APG III system. See [33] for more information on plant surveys.

To assess local bird diversity, 15-min point counts from the center of each study plot were conducted. Birds were identified visually or by call. To minimize bias, all point counts were done by one observer (ELN). During the study period, point counts at each study plot were repeated three times: 6–10 a.m., 10 a.m.–2 p.m. and 2–6 p.m. Additionally, point counts were distributed randomly across study plots and carried out only on days without rain or wind. However, due to logistical constraints, only 27 out of the 30 study plots used for plant surveys were investigated for bird diversity. See [7] for more information on bird surveys.

### Forest fragment characteristics

Within-forest characteristics (related to forest disturbance), the overall size of forest fragments, and shifts in the surrounding matrix (related to local loss in forest cover) were expected to differ in the five types of modified forest, with subsequent effects on TD or PD. These fragment characteristics were measured as follows:

First, GPS tracking was used to measure the size and edge length of the sampled scarp (or secondary) forests. The perimeter to area ratio was calculated using the edge length and forest size. Second, within each study plot, the percentage cover of living biomass (i.e., the cumulative cover of trunks, branches, vines, and foliage) was estimated at seven layers (0.0 m, 0.5 m, 1.0 m, 2.0 m, 4.0 m, 8.0 m, and 16 m) in ten 25-m^2^ subplots. A sighting tube was used to estimate canopy cover at ten randomly chosen locations within the plots. Measures of canopy cover were complemented by measurements of the relative light intensity at each center of the ten subplots used for seedling surveys (in relation to a simultaneously measured reference at a nearby unshaded spot; luxmeter ATP LX-332). The means of the characteristics (biomass at each layer, canopy cover, and relative light intensity) were then calculated for each study plot. The Shannon index of the living biomass in the seven layers served as an estimate of vegetation heterogeneity.

To reduce the number of environmental variables in our analyses, a principal components analysis (PCA) was applied to the correlation matrix of the forest fragment characteristics. This matrix included forest size, edge length, perimeter to area ratio, biomass at each layer, canopy cover, relative light intensity, and vegetation heterogeneity. Two binary dummy variables were also included: (1) scarp or secondary forest and (2) surrounding matrix, natural or agricultural (S1 Table). Scree plots and the broken-stick method were used to estimate the principal components to be retained for further analyses [36]. The first two principal components, accounting for 51.7% (PC1) and 15.9% (PC2) of the environmental variance across the study plots, were thus retained (S2 Table). Factor loadings indicated that PC1 was mainly related to decreasing canopy cover and lower biomass at high layers as well as increases in biomass at low layers and a shift from scarp to secondary forests. PC2 was mainly related to fragment size and edge length and to a shift from a natural to an agricultural matrix (S2 Table). Therefore, in the following, PC1 and PC2 are referred to, respectively, as ‘forest disturbance’ and ‘forest loss’ and they are used to explain the variations in the TD and PD of both the taxa and the successive plant life stages across the study plots.

### Phylogenies

For plants, a phylogenetic super-tree of all mapped plant species (irrespective of their life stage) was generated with the software Phylomatic, version 3.0 [37]. The software prunes an online-available mega-tree (http://phylodiversity.net/phylomatic) of all but the requested plant species. The resulting phylogenetic tree has, however, no defined branch lengths. Branch lengths were adjusted using the ‘bladj’ command. The ‘bladj’ algorithm uses an ‘ages’ file that provides age estimates of specific nodes in the phylogeny and then evenly distributes the remaining undated nodes between these estimates. In this study, an updated and recently published ‘ages’ file (agescl3; [38]) was used that best matched the internal node names of the most recent mega-tree (R20120829; Markus Gastauer, pers. comm.). Additionally, Bayesian polytomy resolution in BEAST was applied [39]. Following [40], we used a constant rate birth-death model and *a priori* constraints on the tree topology from the supplemental R-script of [40]. In total, 11,111,000 permutations were run, with the trees sampled at every 1000th permutation and the first 10% discarded as burn-in, thus yielding a posterior distribution of 10,000 trees. We then calculated a patristic distance matrix of each tree and subsequently averaged across the 10,000 distance matrices to gain a mean phylogenetic distance matrix. This mean distance matrix thus corresponded to the mean phylogenetic distribution of the plant species across the 10,000 sampled trees and was used as the plant species phylogeny for all subsequent analyses.

For birds, a recently published global phylogeny of bird species was used [41]. Here, phylogenetic trees sampled from a pseudo-posterior distribution were downloaded from http://birdtree.org. Five thousand trees (each based on the ‘Hackett’ backbone and covering 9993 species) were downloaded, with each one then pruned to the bird species recorded in our survey. Next, a mean phylogenetic distance matrix was calculated that was used for all subsequent analyses.

### Calculation of diversity components

Diversity components were calculated using a recently proposed framework [42] that partitions species diversity into independent α-, β-, and γ-components, using Rao’s quadratic entropy index [43]. Presently, the Rao index is the only measure of species diversity that can be used to investigate its different facets (e.g., TD and PD) within the same mathematical framework [42]. The index also incorporates information on the relative abundances of species and is therefore highly suitable to detect changes in the taxonomic or phylogenetic composition of species communities across environmental gradients. We used the corrected form of the Rao index as presented in [42] and an additional correction for measures of β-diversity values as proposed in [44]. Multiplicative partitioning was then used to separate regional species diversity (γ) into within-community (α) and among-communities (β) components. Here, γ-diversity was defined as given by equation 1:

$$\;\gamma \;=\;\sum_{i\;=\;1}^{S}\sum_{j\;=\;1}^{S}d_{ij}P_{i}P_{j}$$

where *S* is the total number of species in the study region, *P*_*i*_ and *P*_*j*_ the relative abundances of species *i* and *j* in the region, and *d*_*ij*_ the dissimilarity (taxonomic or phylogenetic) between each pair of species *i* and *j*. Similarly, α-diversity was defined by equation 2:

$$\;\alpha \;=\;\sum_{i\;=\;1}^{S}\sum_{j\;=\;1}^{S}d_{ij}P_{i}P_{j}$$

where *S* is the total number of species within a given community, *P*_*i*_ and *P*_*j*_ the relative abundances of species *i* and *j*, and *d*_*ij*_ the dissimilarity (taxonomic or phylogenetic) between each pair of coexisting species *i* and *j*.

To calculate pairwise β-diversity between study plots, α and γ were calculated for each pair of samples, i.e., study plots. Pairwise β-diversity was then multiplicatively partitioned as given by equation 3:

$$\beta \;=\;\left(\gamma_{pair}-\alpha_{mean}\right)÷\gamma_{pair}\times 100$$

where *γ*_*pair*_ is the total species diversity of the two study plots, and *α*_*mean*_ the mean local diversity of the pair.

Local α-diversity (Equation 2) and pairwise β-diversity (Equation 3) components were calculated for both the TD and the PD of species communities. For PD, we used a distance matrix of the recorded species from patristic distances within their respective phylogenies. Calculations were undertaken for each species group (adult trees, saplings, seedlings, and birds).

A null model analysis was then applied to calculate standardized effect sizes (SES) of PD estimates to investigate whether the observed PD values differed from those that would be expected under random community assembly following equation 4:

$$SES\;=\;\left(OBS-mean\left(EXP\right)\right)÷sdev\left(EXP\right)$$

where *OBS* are the observed values of the α- or β-diversity components of PD, and *EXP* are the similarly calculated values from a null model distribution. To obtain a distribution of expected values under random community assembly, we shuffled the taxa labels across the tips of the species’ phylogenies, calculated the expected α- and β-diversities, and repeated this process 3000 times. Importantly, for plant species, the full regional phylogeny was used for the null model, i.e., a combination of the phylogenetic trees of adult trees, saplings, and seedlings. Thus, our null model allowed for the full phylogenetic variation across all successive plant life stages. In other words, a given plant species could occupy any position in the phylogeny, whether it was recorded as an adult tree, a sapling, or a seedling. Moreover, all recorded tree species were included in this phylogeny, i.e., also those species that were recorded in the three study plots where no point counts of birds had been conducted. In the following, we refer to the calculated SES as α-PD and β-PD. Note that, based on our null model, SES were calculated only for the PD of the taxa and not for the α-TD or β-TD components. Furthermore, note that α-PD could not be calculated for the saplings in one study plot. Here, only one sapling species was recorded and the standard deviation of expected values was therefore zero. A statistically significant positive departure of SES from the null assumption (overdispersion) can indicate effects of limiting similarity on community assembly [22], and a significant negative departure (underdispersion) the effects of environmental filtering [23,45]. Significant overdispersion and underdispersion of SES were calculated from the percentage of cases in which the observed values were, respectively, greater or less than the expected values at α = 0.025 [46].

### Statistical analyses

#### Alpha-diversity components

First, differences in α-TD and α-PD among the three successive plant life stages (adult trees, saplings, seedlings) were investigated. Here, repeated measures ANOVAs were fitted with study plot as random effect. Pairwise differences between successive life stages were investigated with Tukey tests, using the Bonferroni correction for multiple comparisons. Our measures of PD for plants and for birds were based on effect sizes that derived from different null models and thus were not directly comparable. Consequently, the variations in α-PD between plant life stages and birds were not directly compared in the same model. However, correlations in the variations of α-TD and α-PD between plants and birds were investigated.

Second, the effects of forest disturbance and forest loss on α-TD and α-PD were examined, with a specific focus on the differences in the responses of the successive plant life stages and between plants and birds. For the former, linear mixed-effects models were fitted with study plot as random effect. These models additionally included the interactions of predictors with successive life stages, which allowed an assessment of the differences in the responses of adult trees, saplings, and seedlings to environmental variation. Furthermore, the UTM coordinates (easting and northing) of the study plots were included to account for spatial trends in the data. For birds, multiple linear regressions were fitted, using forest disturbance, forest loss, and the UTM coordinates as predictors. In statistical notation, the full model for plants can be expressed by equation 5:

$$Diversity\;component\;\sim \;life\;stage\;\times \left(forest\;disturbance+forest\;loss+easting+northing\right)+(1|study\;plot)$$

and the full model for birds by equation 6:

Diversity component ~ forest disturbance+forest loss+easting+northing

where in each model the diversity component is either α-TD or α-PD.

Model dredging was used to retain the models with the most likely combinations of predictors. In this approach, a model set based on a global model (Equation 5 or Equation 6) is created that includes all possible combinations of predictors, up to a model including only the intercept [47]. Accordingly, we created a model set for each of the four global models and compared the models within a given set using Akaike’s information criterion for small sample sizes (AIC_c_). Models with ΔAIC_c_ < 2.0 are generally regarded as equivalently likely [48]. Since we found multiple models to be equivalently likely, except for the model set on the α-TD of birds (α-TD of plants: five models; α-PD of plants: eight models; α-PD of birds: two models), we used model averaging on the models with ΔAIC_c_ < 2.0 in a given model set [47]. Model averaging calculates a weighted mean of parameter estimates, with weights according to the likelihood of each model [47]. Furthermore, the Akaike weights of the predictors in the averaged model were calculated as the summed Akaike weights of each model with ΔAIC_c_ < 2.0 in which a given predictor appeared. Akaike weights range between 0 and 1, with higher values indicating a higher relative importance. All models were fitted using maximum likelihood to enable comparisons with AIC_c_ [47]. With the exception of life stage, all predictors were Z-transformed to facilitate comparisons of effect sizes.

As stated above, the Rao index is presently the only diversity measure that can be used to investigate different facets of species diversity within the same mathematical framework [42]. However, to ensure that our results were not biased by our choice of diversity measure, the data were re-analyzed using mean pairwise phylogenetic distances (MPD, see for example [15,16]) instead of the Rao α-PD as the dependent variable. Similar to the Rao α-PD, the SES of the MPD were calculated by shuffling the taxa labels across the phylogenies (999 permutations). The analysis generally supported the results on the effects of forest disturbance, forest loss and spatial trends on α-PD, thus confirming that our findings were not biased by our choice of phylogenetic measure (S3 Table).

#### Beta-diversity components

Partial mantel tests were used to investigate changes in the β-TD or β-PD of the successive plant life stages and of birds in response to increasing forest disturbance and forest loss across study plots. Mantel correlations between pairs of study plots were conditioned on an Euclidean plot-plot distance matrix to account for spatial trends. Variation partitioning was applied to assess the variance in diversity components that was explained by forest disturbance, forest loss, and spatial trends [49]. Following [50], spatial eigenvectors were used to account for spatial trends in this analysis. Spatial eigenvectors are particularly suitable for partitioning the species diversity of differently mobile organisms as they capture spatial trends across a range of different spatial scales [51]. The orthogonal spatial eigenvectors of an Euclidean distance matrix of our study plots were derived using principal coordinates of neighbor matrices analysis (PCNM). Eigenvectors that significantly explained the β-TD or β-PD of species groups were then determined using forward selection based on a RDA, employing the double-stop criterion at α = 0.050 [52].

#### Testing for phylogenetic uncertainties

In this study, we used mean phylogenetic distance matrices of plants and birds to investigate the variations in the α-PD and β-PD of taxonomic groups and life stages in response to forest modifications. However, this approach may mask phylogenetic variation across the posterior distribution of trees. To confirm that our statistical approach was not biased by phylogenetic uncertainties, we selected 1000 trees from the posterior distributions of plants and birds and calculated 1000 estimates of α-PD and β-PD based on each of these trees for all species groups. The aforementioned statistical analyses (mixed-effects models with model averaging, partial mantel tests) were then repeated for each of these estimates. The distributions of test statistics showed that the results were similar to those obtained from the mean phylogenetic distance matrices (S4 Table; S5 Table). Therefore, we concluded that our findings were robust even in the case of phylogenetic uncertainties. All statistical analyses were done in the ‘R’ environment [53], using packages ‘lme4’ [54], ‘MuMIn’ [55], ‘packfor’ [56], and ‘picante’ [57].

## Results

The 3637 plant individuals that were sampled covered 166 tree species. Among these individuals, 2150 were adult trees (species richness per study plot [mean ± SD]: 16.6 ± 6.68), 691 were saplings (8.07 ± 3.97), and 796 were seedlings (7.74 ± 4.69). For birds, 738 individuals covering 85 species (13.2 ± 4.13) were recorded.

### Alpha-diversity components

**Fig 2:**
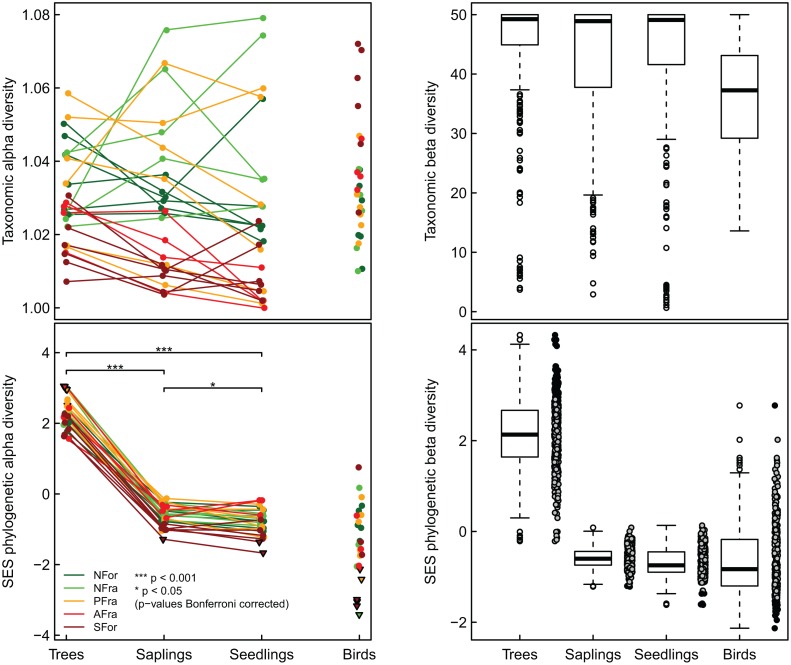
Differences in the taxonomic and phylogenetic α- and β-diversity between species groups. Significant differences of between-diversity facets for the successive life stages of trees (after Bonferroni correction) are indicated by stars. No significant differences were found for taxonomic α-diversity. Different colors show the five types of forest modification (NFor = large natural scarp forests; NFra = natural scarp forest fragments; PFra = scarp forest fragments within eucalyptus plantations; AFra = scarp forest fragments within sugarcane plantations; SFor = secondary forests). The β-diversity facets are shown as boxplots; for phylogenetic β-diversity, the raw data are presented as well. Note that phylogenetic diversity (gray circles) is based on standardized effect sizes (SES). Triangles (α-diversity) and black circles (β-diversity) indicate significant deviation from the null expectation.

**Fig 3:**
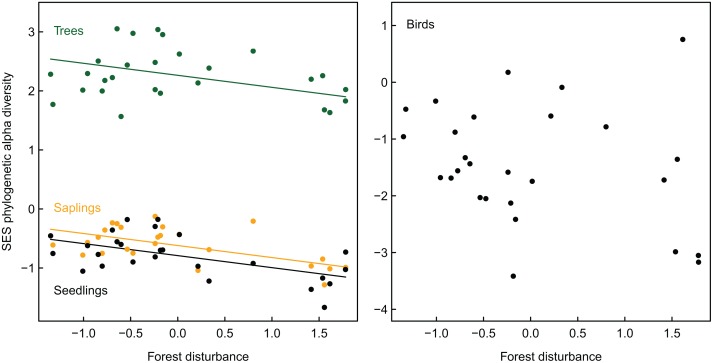
Phylogenetic α-diversity of plant life stages and of birds with increasing forest disturbance. The coefficients (lines) after model averaging are shown, as are the raw data (circles) of species groups (green color: trees; yellow: saplings; black: seedlings and birds). Note that phylogenetic diversity is based on standardized effect sizes (SES) and that the scaling of the y-axes differs. Forest loss has been scaled to zero mean and unit variance.

There were no differences in α-TD among the successive plant life stages, i.e., adult trees, saplings, and seedlings (repeated measures ANOVA: F_2,52_ = 1.32; p = 0.275; Fig. 2). By contrast, the α-PD strongly differed (repeated measures ANOVA: F_2,50_ = 1166; p < 0.001; Fig. 2), decreasing with the decreasing age of the trees, from adult trees (2.27 ± 0.0817; mean ± SE throughout) to saplings (−0.617 ± 0.0586), to seedlings (−0.795 ± 0.0727). For birds, the mean α-PD was −1.45 ± 0.200. In five study plots the α-PD of the adult trees was overdispersed, whereas for saplings and seedlings either it did not differ from random community assembly or it was significantly underdispersed (one and two study plots, respectively). Bird communities were significantly underdispersed in six of the study plots (Fig. 2). The α-TD of birds correlated negatively with the α-TD of all three successive plant life stages (Pearson’s correlations; adults: r = −0.605; p < 0.001, saplings: r = −0.578 p = 0.00158, seedlings: r = −0.545; p = 0.00329; S1 Fig.) whereas there was no correlation between the α-PD of birds and that of any of these life stages (adults: r = −0.276; p = 0.164, saplings: |r| = < 0.001; p = 0.996, seedlings: r = −0.219; p = 0.282; S1 Fig.). Increasing forest disturbance did not significantly affect the α-TD of any plant life stage but it did increase the α-TD of birds (Table 1a). For both plants and birds, forest loss had no effect on either the α-TD or the α-PD (Table 1a, 1b). However, increasing forest disturbance led to a significant loss in α-PD across all plant life stages but had no effect on the α-PD of birds (Table 1b, Fig. 3). Although the latter were altered by spatial trends, the effects on plants were not significant (Table 1b).

**Table 1:** Effects of forest disturbance, forest loss, and spatial trends (easting and northing of the study plots) on the taxonomic and phylogenetic α-diversity of species groups.

	Estimate	SE	Adjusted SE	Z	P	Akaike weight
**(a) Taxonomic α-diversity**						
**Plants**						
Intercept	1.03	0.00261	0.00266	387	< 0.001	
Forest disturbance	-0.00758	0.00392	0.00396	1.91	0.0557	0.67
Easting	0.0190	0.0130	0.0131	1.45	0.147	0.65
Northing	-0.0172	0.0146	0.0147	1.17	0.241	0.59
**Birds**						
Intercept	1.03	0.00236		439^a^	< 0.001	
**Forest disturbance**	**0.0112**	**0.00240**		**4.68** ^**a**^	**< 0.001**	
**(b) Phylogenetic α-diversity**						
**Plants**						
Intercept (= adult trees)	2.26	0.0561	0.0570	39.7	< 0.001	
*Successive life stage*						1.0
**Saplings**	**-2.88**	**0.0682**	**0.0694**	**41.6**	**< 0.001**	
**Seedlings**	**-3.05**	**0.0690**	**0.0702**	**43.5**	**< 0.001**	
**Forest disturbance**	**-0.204**	**0.0596**	**0.0602**	**3.39**	**< 0.001**	**1.0**
Forest loss	0.0927	0.0522	0.0530	1.75	0.0802	0.51
Easting	-0.0188	0.205	0.207	0.0910	0.928	0.32
Northing	0.188	0.146	0.147	1.28	0.201	0.65
*Successive life stage × Northing*						0.18
Saplings ×Northing	-0.114	0.0670	0.0682	1.67	0.0941	
Seedlings × Northing	-0.115	0.0671	0.0683	1.68	0.0923	
**Birds**						
Intercept	-1.45	0.171	0.180	8.04	< 0.001	
Forest loss	0.337	0.213	0.224	1.50	0.133	0.49
**Northing**	**-0.643**	**0.219**	**0.228**	**2.82**	**0.00482**	**1.0**

Separate models were calculated for (a) plants and (b) birds. The coefficients from model averaging of equivalently likely models (see Methods) are shown. Akaike weights yield information on the relative importance of multiple predictors per model. Statistically significant predictors (p < 0.050) are shown in boldface type. With the exception of life stage, all predictors were Z-transformed to facilitate comparisons of effect sizes.

^a^ Note that these are t-values from the single “best” multiple linear regression model; no model averaging was necessary and Z-values are reported for averaged models only.

### Beta-diversity components

**Fig 4:**
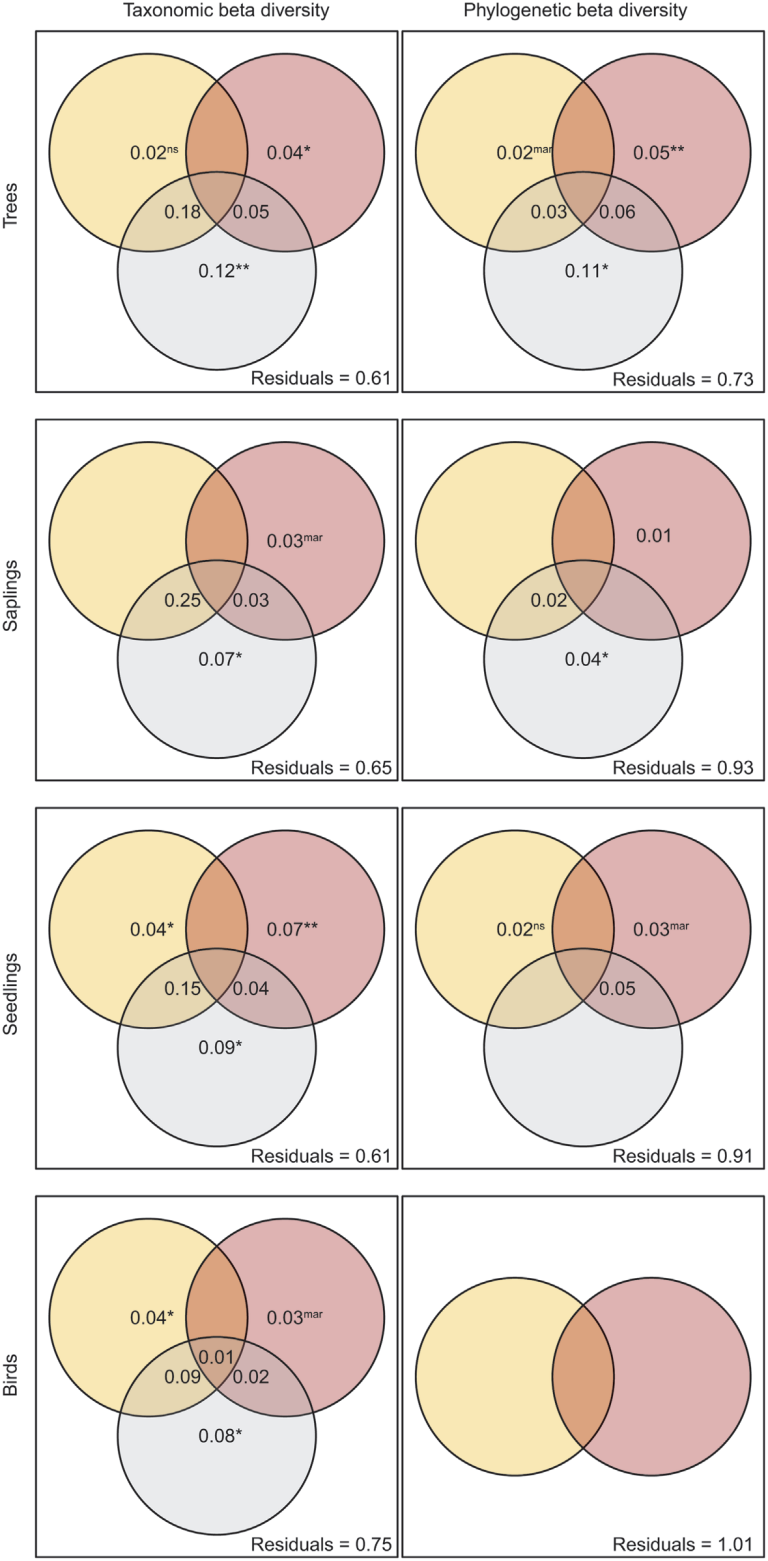
Variation partitioning of taxonomic and phylogenetic β-diversity of species groups. Venn diagrams illustrate the variation explained by forest disturbance (yellow), forest loss (red), and spatial trends (gray). The combined and separate effects of environmental variables are depicted. Values < 0.010 are not shown. No spatial eigenvectors significantly described the variation in the phylogenetic β-diversity of birds. Statistical significance levels are indicated for separate effects of environmental variables (significances cannot be calculated for joint effects): ** p < 0.010; * p < 0.050; ^mar^ = p < 0.100; ^ns^ = not significant.

Partial mantel tests showed that with increasing differences in forest disturbance between pairs of study plots the β-TD of plants and of birds increased as well (Table 2). Comparable positive correlations were determined for differences in forest loss and the β-TD of adult trees and seedlings, but not of saplings and birds (Table 2). By contrast, the effects of site-specific differences in forest disturbance and forest loss on the β-PD of species groups were weak (Table 2). The results from variation partitioning confirmed those from the partial mantel tests (Fig. 4). The cumulative variance in β-TD explained by forest disturbance, forest loss, and spatial trends was in the range of 35–39% among successive plant life stages and was 25% for birds. While the separately explained variances of environmental variables were comparable in size, joint effects were strongest for forest disturbance and spatial trends (Fig. 4). Unlike the β-TD, the residual variation in the β-PD of species groups after variation partitioning remained considerably higher (explained variance: plants = 7–27%; birds = 0%) and separate effects of forest disturbance, forest loss, and spatial trends were in many cases not statistically significant (Fig. 4).

**Table 2:** Results from partial mantel tests investigating the effects of forests disturbance and forests loss on the taxonomic and phylogenetic β-diversity among species groups.

	Taxonomic β-diversity		Phylogenetic β-diversity	
	Forest disturbance	Forest loss	Forest disturbance	Forest loss
Adult trees	0.424***	0.175**	-0.286ns	0.101^ns^
Saplings	0.490***	0.0962^mar^	0.111^mar^	0.0201^ns^
Seedlings	0.436***	0.186**	-0.00200^ns^	0.187*
Birds	0.291**	-0.0354^ns^	0.0272^ns^	-0.153^ns^

Correlations were conditioned on a spatial distance matrix of the study plots. The correlation coefficients are shown; asterisks indicate the significance level.

*** p < 0.001;

** p < 0.010;

* p < 0.050;

^mar^ = p < 0.100;

^ns^ = not significant.

## Discussion

In this study, different degrees of forest disturbance and forest loss imposed contrasting effects on the taxonomic and phylogenetic α- and β-diversity of successive plant life stages and birds in a subtropical landscape. The α-TD of plants correlated negatively with that of birds for all successive life stages, whereas for α-PD there was no correlation in the SES between taxa. Forest disturbance was not related to the α-TD of plants; however, the α-TD of birds increased with increasing disturbance. By contrast, the α-PD of all plant life stages decreased with increasing forest disturbance, whereas the α-PD of birds was unaffected. At the landscape scale, the taxonomic, but not the phylogenetic β-diversity of both taxa was well predicted by variations in forest disturbance and forest loss. Finally, in contrast to adult trees, the PD of saplings and seedlings showed signs of contemporary environmental filtering, indicating a time-lag in the responses of tree species to present-day changes in landscape structure.

### Alpha-diversity components

There was no congruence in the α-TD and α-PD of species groups in response to forest disturbance or forest loss. Contemporary forest disturbance did not reduce the α-TD of any plant life stage. In our study, forest disturbance was mainly related to a loss of canopy cover, increasing biomass at lower forest layers, and an overall loss in vegetation heterogeneity. Generally, such changes in forest structure have been shown to threaten tree diversity, either directly, via human-induced activities (e.g., logging; [58]), or indirectly, because of a loss of animal-mediated processes related to plant regeneration (e.g., pollination and seed dispersal of plants; [59]). However, in the study region these threats were not evident from our results on the variation in the α-TD of the plants. Previous studies in the same region indicated a high functional connectivity of natural and modified forest fragments, as suggested by the frequent movements of seed dispersers between fragments [60] and the weak effects of the surrounding matrix on forest communities [32,33]. Moreover, given the natural fragmentation of scarp forests and the frequent vegetation changes that occurred over the Pleistocene, these forest communities may have evolved such that they are highly resistant to contemporary forest disturbance and forest loss [30,61–63]. In fact, the α-TD of birds increased in disturbed forests, a finding consistent with studies showing that in birds this type of diversity can be sustained in human-modified tropical landscapes [64]. Nonetheless, previous studies in the region showed the frequent loss of forest-specialist birds [7,65], which suggests that for these species the negative effects of forest disturbance can be masked by turnovers in community composition [7,66,67]. Interestingly, in addition to the contrasting responses across species groups to forest modification, we found negative correlations between within-fragment measures of plant and bird TD. In other words, forest fragments with a high plant diversity supported a low bird diversity and vice versa. Thus, the conservation of species TD in the study region seems to depend on the preservation of a heterogeneous landscape composition. Here, even modified forests can make valuable contributions [7,32,68].

Forest disturbance did not affect the α-TD of plants but it reduced the α-PD across their successive life stages. Amongst others, life-history states that make species more susceptible to local extinction include a slow growth rate, high specialization on mutualistic partners, and high habitat or resource specialization [65,69–71]. Accordingly, [72] show negative effects of forest modification on slow-growing and late-successional tree species and increases in the offspring of fast-growing and early-successional species in our study region. Thus, phylogenetic signal in these or other plant response traits related to forest disturbance may help to explain the observed changes in the composition of tree communities and losses of α-PD of plants with forest disturbance.

Contrary to our expectations and to the reported decline in the α-PD of trees in a fragmented Brazilian Atlantic forest [69], forest loss in the study region had no effect on the α-PD of any species group. Potentially, contemporary and ongoing forest loss in our study was only partially covered by the selected principal component. Furthermore, this result seems to contradict what might be expected from species—area relationships. However, the relationships between the PD of species and areas (e.g., forest cover) are poorly understood and may diverge from more traditional TD—area relationships. For example, the spillover of distantly related species from the agricultural matrix may have buffered the α-PD within forest fragments [24]. Also, bird communities in our study region were found to be overall robust to forest modification (despite losses in forest specialists), with bird individuals frequently dispersing across the agricultural matrix [7,60]. The forest fragments in the region support a high functional diversity of birds as well as associated food plants [68], which would at least in part explain the weak effects of forest disturbance and forest loss on the α-PD of the bird species identified in this study.

### Beta-diversity components

In contrast to our results on the α-diversities of species groups, forest disturbance and forest loss imposed overall strong positive effects on β-TD, but only weak relationships with β-PD. Forest disturbance and the loss of forest cover thus amplified a taxonomic species turnover across forest communities, shaping a mosaic of forest biodiversity. Interestingly, local environmental conditions and spatial effects explained more of the variation in the β-diversities of plant life stages than in the β-diversity of birds. Thus, different than in sessile plants, the mobility of many bird species in the region seems to mitigate the species turnover resulting from local forest modification or isolation by distance [60]. The weaker relationships between forest modification and the β-PD of species groups indicated that the taxonomic species turnover did not follow from the phylogenetic relationships of the involved species. A previous study [73] likewise found that the environmental conditions in six temperate and tropical forest communities strongly shaped the α-diversity of tree communities, yet poorly predicted β-PD. Here, the lack of a phylogenetic signal in those functional traits of plant species that may have determined β-PD seemed to be the primary explanation. Similarly, potentially important response traits in our study system had a weak phylogenetic signal (see Discussion on the α-PD of taxa). Divergent patterns in β-TD and β-PD across different environments can occur against a background of taxonomic species turnover of communities composed of small-range species, while simultaneously these communities are phylogenetically randomly assembled across different geographic distances [25]. Correspondingly, effects of spatial trends on forest communities in our study were strongest for β-TD and considerably weaker for β-PD.

### Time-lags in species responses to forest modifications?

Despite the differences in the effects of forest disturbance and forest loss on the α- and β-PD of all plant life stages, marked losses in both diversity facets were noted from adult trees to younger life stages. While in some cases the PD of adult trees showed signs of overdispersion, indicative of competitive interactions within tree assemblages, the PD of saplings and seedlings suggested either random community assembly or underdispersion. As noted above, underdispersion may reflect the contemporary effects of environmental filtering on the PD of younger plant life stages [20,21]. This finding suggests that the scarp forest fragments in this study are subject to an extinction debt [9,12]. However, it should be noted that scarp forests have been naturally fragmented since the last glacial maximum [30,61]. Specifically, tree species that are endemic to scarp forests may have evolved adaptations to forest fragmentation that allow them to persist even in an ever more human-shaped forest landscape [62,63]. Moreover, since forest disturbance negatively affected the PD of all plant life stages, it cannot have been the sole driver of the reduced PD in younger life stages. We therefore caution against the conclusion that an extinction debt of forest communities is the only explanation for the higher α- and β-PD of adult trees vs. the other species groups. Still, time-lags in species responses to forest disturbance and forest loss are of particular concern in forest ecosystems. For example, in Brazilian Atlantic forests there was a lag time in the responses of Myrtaceae species to a loss of forest cover [12]. Another study found that time-lags were most pronounced for tree species and that the diversity of mobile taxa (birds, frogs, and mammals) was more closely related to contemporary patterns in forest distribution [11]. Although the effects of forest loss on historically fragmented landscapes, such as our study region, are probably different from those on recently fragmented landscapes, such as the Brazilian Amazon [62], the α- and β-PD of birds in our study was the lowest among all species groups and frequently seemed to be subject to local environmental filtering. Following the precautionary principle and to avoid potential future losses in the PD of forest communities, we call on conservation managers and landowners to ensure the preservation of forested areas, especially unprotected scarp forest fragments that may act as refuges and stepping stones within the agricultural matrix.

## Supporting Information

S1 Fig Correlations of taxonomic and phylogenetic α-diversity between life stages of plants and birds across the 27 study plots. (EPS)

S1 Table Matrix of environmental variables recorded across the 27 study plots. (DOC)

S2 Table Results from the principal components analysis on the environmental variables of study plots. Shown are factor loadings of environmental variables, as well as the explained and cumulative proportion of environmental variance across components. Brocken-stick analysis was used to derive the number of components to be retained for further analysis (see Methods). The first principal components (PC1 and PC2) were retained for further analyses.
(DOC)

S3 Table Using mean pairwise phylogenetic distances (MPD) instead of the Rao index for phylogenetic α-diversity gives similar results. Shown are effects of forest disturbance, forest loss and spatial trends on phylogenetic α-diversity of species groups. Note the similarity in effect directions and effect sizes to those based on the Rao index shown in Table 1.
(DOC)

S4 Table Means of test statistics from 1000 repetitions of statistical analyses (see Methods) on variation in phylogenetic α-diversity of life stages of adult trees, saplings and seedlings and of birds. Each repetition was based on one of 1000 phylogenetic trees per species group. The last column gives information on the number of analyses in which a predictor appeared in the final averaged model. Note that predictors that appeared in each analysis (n = 1000) also appear in Table 1b, and that effect sizes are comparable to those based on mean phylogenetic distance matrices of phylogenetic trees from the posterior distributions (Table 1b). Statistically significant predictors (p < 0.050) are shown in boldface type.
(DOC)

S5 Table Results of 1000 partial mantel tests on changes in phylogenetic beta diversity of life stages of plants and of birds. Each test was based on one of 1000 phylogenetic trees per species group. Correlations were conditioned on a spatial distance matrix of study plots. Shown are means and standard deviations of Pearson’s correlation coefficient and p-values. Correlations with p_mean_ < 0.050 are shown in boldface type. Note that effect sizes are comparable to those from the analysis based on a mean phylogenetic distance matrix instead of the posterior distribution (Table 2).
(DOC)
