# Supplementary material for: Contrasting Taxonomic and Phylogenetic Diversity Responses to Forest Modifications: Comparisons of Taxa and Successive Plant Life Stages in South African Scarp Forest
Source: PLoS One. 2015 Feb 26;10(2):e0118722. doi: 10.1371/journal.pone.0118722 (PMC4342016; doi:10.1371/journal.pone.0118722)
Supplement: S1 Table — (DOC) [file pone.0118722.s002.doc]

Table S1. Matrix of environmental variables recorded across the 27 study plots.

| **Study plot** | **Forest modification type^a^** | **Forest size [ha]** | **Forest edge length [m]** | **Perimeter to area ratio** | **Living biomass 0 m [%]** | **Living biomass 0.5 m [%]** | **Living biomass 1.0 m [%]** | **Living biomass 2.0 m [%]** | **Living biomass 4.0 m [%]** | **Living biomass 8.0 m [%]** | **Living biomass 16 m [%]** | **Canopy cover [%]** | **Relative light intensity [%]** | **Vegetation heterogeneity [Shannon-Index]** | **Scarp forest [yes/no]** | **Matrix natural [yes/no]** |
| --- | --- | --- | --- | --- | --- | --- | --- | --- | --- | --- | --- | --- | --- | --- | --- | --- |
| 1.1 | NFor | 130 | 17566 | 135 | 20.0 | 29.0 | 29.0 | 31.5 | 52.0 | 76.5 | 2.0 | 86.8 | 0.64 | 1.720 | 1 | 1 |
| 1.2 | NFor | 130 | 17566 | 135 | 34.5 | 46.5 | 30.5 | 39.0 | 59.0 | 53.5 | 44.5 | 91.3 | 0.14 | 1.923 | 1 | 1 |
| 1.3 | NFor | 822 | 38395 | 47 | 14.0 | 23.0 | 23.0 | 37.5 | 51.0 | 57.5 | 64.5 | 93.0 | 1.05 | 1.832 | 1 | 1 |
| 1.4 | NFor | 822 | 38395 | 47 | 26.0 | 34.5 | 31.0 | 31.0 | 45.5 | 55.5 | 1.0 | 90.0 | 0.27 | 1.777 | 1 | 1 |
| 1.5 | NFor | 822 | 38395 | 47 | 36.0 | 46.0 | 30.0 | 54.5 | 69.0 | 51.5 | 19.0 | 80.3 | 3.17 | 1.880 | 1 | 1 |
| 1.6 | NFor | 822 | 38395 | 47 | 15.0 | 21.0 | 23.0 | 26.5 | 45.0 | 79.5 | 64.5 | 97.8 | 0.76 | 1.785 | 1 | 1 |
| 2.2 | NFra | 0.54 | 298 | 552 | 20.5 | 49.0 | 35.5 | 29.0 | 44.0 | 51.5 | 9.5 | 83.5 | 4.65 | 1.845 | 1 | 1 |
| 2.3 | NFra | 2.8 | 859 | 307 | 21.0 | 32.5 | 23.0 | 24.0 | 46.5 | 73.5 | 23.5 | 86.3 | 0.77 | 1.833 | 1 | 1 |
| 2.4 | NFra | 7.25 | 1399 | 193 | 64.5 | 74.5 | 40.0 | 41.0 | 49.0 | 56.0 | 34.0 | 76.8 | 0.82 | 1.912 | 1 | 1 |
| 2.5 | NFra | 7.15 | 1732 | 242 | 13.0 | 26.0 | 28.5 | 28.5 | 52.5 | 79.5 | 59.5 | 94.0 | 0.73 | 1.808 | 1 | 1 |
| 2.6 | NFra | 0.61 | 445 | 730 | 31.5 | 45.5 | 34.5 | 43.0 | 57.5 | 62.5 | 18.5 | 81.3 | 0.42 | 1.886 | 1 | 1 |
| 3.1 | PFra | 12.63 | 6038 | 478 | 49.5 | 56.5 | 45.0 | 42.5 | 51.0 | 50.0 | 0.0 | 58.3 | 0.83 | 1.788 | 1 | 0 |
| 3.2 | PFra | 12.63 | 6038 | 478 | 53.5 | 62.5 | 31.5 | 37.5 | 40.5 | 48.5 | 28.5 | 72.3 | 0.54 | 1.912 | 1 | 0 |
| 3.3 | PFra | 12.63 | 6038 | 478 | 36.5 | 47.0 | 34.5 | 40.5 | 54.5 | 55.0 | 15.0 | 67.5 | 3.81 | 1.888 | 1 | 0 |
| 3.4 | PFra | 12.63 | 6038 | 478 | 50.0 | 57.0 | 44.0 | 60.5 | 38.5 | 16.0 | 5.0 | 77.8 | 3.52 | 1.788 | 1 | 0 |
| 3.5 | PFra | 12.63 | 6038 | 478 | 36.0 | 44.5 | 32.5 | 44.0 | 34.0 | 36.5 | 50.0 | 86.5 | 0.35 | 1.934 | 1 | 0 |
| 3.6 | PFra | 12.63 | 6038 | 478 | 66.0 | 71.5 | 52.0 | 39.0 | 39.0 | 9.5 | 0.0 | 59.0 | 0.89 | 1.673 | 1 | 0 |
| 4.2 | AFra | 5.57 | 950 | 171 | 29.5 | 43.5 | 50.0 | 61.5 | 37.5 | 38.0 | 38.0 | 80.5 | 0.10 | 1.921 | 1 | 0 |
| 4.3 | AFra | 5.2 | 1192 | 229 | 14.0 | 18.5 | 27.0 | 80.5 | 41.5 | 49.5 | 30.0 | 92.0 | 0.28 | 1.798 | 1 | 0 |
| 4.4 | AFra | 2.28 | 675 | 296 | 21.0 | 26.5 | 36.5 | 79.0 | 40.0 | 41.5 | 58.0 | 87.5 | 0.14 | 1.861 | 1 | 0 |
| 4.5 | AFra | 10.97 | 2415 | 220 | 19.0 | 23.5 | 25.5 | 91.5 | 43.0 | 58.0 | 62.5 | 90.5 | 0.31 | 1.810 | 1 | 0 |
| 5.1 | SFor | 10.01 | 3445 | 344 | 92.0 | 98.0 | 52.5 | 26.0 | 26.0 | 24.5 | 0.0 | 33.0 | 4.49 | 1.624 | 0 | 0 |
| 5.2 | SFor | 5.61 | 2053 | 366 | 80.5 | 84.0 | 39.5 | 30.0 | 25.5 | 0.0 | 0.0 | 50.8 | 29.53 | 1.492 | 0 | 0 |
| 5.3 | SFor | 0.23 | 288 | 1252 | 69.0 | 79.0 | 24.0 | 18.5 | 40.5 | 20.5 | 0.0 | 33.3 | 11.6 | 1.633 | 0 | 0 |
| 5.4 | SFor | 0.046 | 81 | 1761 | 65.5 | 70.5 | 63.0 | 22.0 | 35.5 | 0.0 | 0.0 | 48.3 | 12.0 | 1.533 | 0 | 0 |
| 5.5 | SFor | 34.03 | 5986 | 176 | 81.5 | 86.0 | 12.0 | 12.0 | 31.5 | 20.0 | 2.0 | 40.3 | 32.5 | 1.537 | 0 | 0 |
| 5.6 | SFor | 34.03 | 5986 | 176 | 69.5 | 76.5 | 39.5 | 32.5 | 18.5 | 16.5 | 7.0 | 30.5 | 33.9 | 1.719 | 0 | 0 |

^a^ Abbreviations of forest modification types: NFor = large natural scarp forests; NFra = natural scarp forest fragments; PFra = scarp forest fragments within eucalyptus plantations; AFra = scarp forest fragments within sugarcane plantations; SFor = secondary forests.
