# Supplementary material for: Contrasting Taxonomic and Phylogenetic Diversity Responses to Forest Modifications: Comparisons of Taxa and Successive Plant Life Stages in South African Scarp Forest
Source: PLoS One. 2015 Feb 26;10(2):e0118722. doi: 10.1371/journal.pone.0118722 (PMC4342016; doi:10.1371/journal.pone.0118722)
Supplement: S2 Table — Shown are factor loadings of environmental variables, as well as the explained and cumulative proportion of environmental variance across components. Brocken-stick analysis was used to derive the number of components to be retained for further analysis (see Methods). The first principal components (PC1 and PC2) were retained for further analyses. (DOC) [file pone.0118722.s003.doc]

**Table S2. Results of a principal components analysis on environmental variables of study plots.** Shown are factor loadings of environmental variables, as well as the explained and cumulative proportion of environmental variance across components. Brocken-stick analysis was used to derive the number of components to be retained for further analysis (see Methods). The first principal components (PC1 and PC2) were retained for further analyses.

|  |  | **Components** | | | | | | | | | | | | | | |
| --- | --- | --- | --- | --- | --- | --- | --- | --- | --- | --- | --- | --- | --- | --- | --- | --- |
| **Factor loadings** | **Factors** | **PC1** | **PC2** | **PC3** | **PC4** | **PC5** | **PC6** | **PC7** | **PC8** | **PC9** | **PC10** | **PC11** | **PC12** | **PC13** | **PC14** | **PC15** |
|  | Forest size | -0.18 | -0.46 | 0.04 | 0.45 | -0.17 | -0.02 | -0.13 | -0.05 | 0.06 | -0.08 | -0.19 | 0.19 | -0.32 | 0.55 | -0.13 |
|  | Forest edge length | -0.18 | -0.47 | 0.03 | 0.44 | -0.06 | 0.04 | -0.11 | -0.14 | 0.05 | -0.03 | 0.09 | -0.02 | 0.35 | -0.58 | 0.22 |
|  | Perimeter to area ratio | 0.19 | 0.17 | -0.45 | -0.08 | -0.65 | 0.03 | -0.33 | -0.02 | 0.21 | 0.03 | -0.18 | 0.31 | 0.02 | -0.14 | 0.05 |
|  |  |  |  |  |  |  |  |  |  |  |  |  |  |  |  |  |
|  | Living biomass 0.0 m | 0.33 | -0.08 | -0.05 | 0.09 | 0.33 | -0.06 | -0.28 | 0.01 | -0.26 | 0.22 | -0.05 | 0.36 | 0.20 | -0.12 | -0.62 |
|  | Living biomass 0.5 m | 0.32 | -0.08 | -0.13 | 0.03 | 0.39 | -0.10 | -0.27 | 0.05 | -0.09 | 0.21 | -0.15 | 0.15 | -0.02 | 0.18 | 0.71 |
|  | Living biomass 1.0 m | 0.16 | 0.27 | -0.37 | 0.52 | 0.06 | -0.40 | 0.32 | 0.26 | -0.02 | -0.31 | 0.25 | 0.05 | 0.07 | 0.05 | 0.00 |
|  | Living biomass 2.0 m | -0.14 | 0.42 | 0.33 | 0.34 | 0.00 | 0.40 | -0.04 | 0.39 | -0.16 | -0.08 | -0.41 | 0.18 | -0.02 | -0.14 | 0.09 |
|  | Living biomass 4.0 m | -0.26 | -0.07 | -0.41 | -0.05 | 0.08 | 0.45 | -0.35 | 0.38 | -0.19 | -0.05 | 0.43 | -0.18 | 0.06 | 0.16 | -0.02 |
|  | Living biomass 8.0 m | -0.31 | -0.07 | -0.09 | -0.33 | 0.10 | -0.05 | 0.10 | -0.18 | -0.32 | -0.54 | -0.07 | 0.53 | 0.18 | 0.03 | 0.12 |
|  | Living biomass 16 m | -0.25 | 0.12 | 0.35 | -0.07 | -0.20 | -0.59 | -0.46 | 0.21 | -0.24 | 0.06 | 0.25 | 0.01 | -0.14 | -0.08 | 0.05 |
|  | Canopy cover | -0.34 | 0.09 | 0.02 | 0.01 | -0.12 | -0.10 | 0.22 | 0.10 | 0.10 | 0.52 | 0.01 | 0.19 | 0.60 | 0.35 | 0.05 |
|  | Relative light intensity | 0.26 | -0.24 | 0.34 | -0.18 | 0.00 | 0.10 | 0.06 | 0.46 | 0.49 | -0.16 | 0.32 | 0.37 | 0.03 | -0.01 | 0.03 |
|  | Vegetation heterogeneity | -0.28 | 0.22 | -0.07 | 0.01 | 0.39 | -0.16 | -0.39 | -0.09 | 0.61 | -0.24 | -0.21 | -0.12 | 0.16 | 0.04 | -0.12 |
|  |  |  |  |  |  |  |  |  |  |  |  |  |  |  |  |  |
|  | Scarp or secondary forest | -0.32 | 0.19 | -0.13 | 0.10 | 0.22 | 0.10 | 0.11 | -0.22 | 0.18 | 0.34 | 0.32 | 0.42 | -0.49 | -0.22 | 0.03 |
|  | Matrix natural or anthropogenic | -0.24 | -0.32 | -0.31 | -0.21 | 0.10 | -0.24 | 0.22 | 0.52 | 0.03 | 0.15 | -0.42 | -0.05 | -0.20 | -0.27 | -0.08 |
|  |  |  |  |  |  |  |  |  |  |  |  |  |  |  |  |  |
|  |  |  |  |  |  |  |  |  |  |  |  |  |  |  |  |  |
| **Proportion of variance** | Explained | 0.52 | 0.16 | 0.09 | 0.07 | 0.05 | 0.03 | 0.03 | 0.02 | 0.01 | 0.01 | 0.01 | 0.00 | 0.00 | 0.00 | 0.00 |
|  | Cumulative | 0.52 | 0.68 | 0.76 | 0.83 | 0.88 | 0.91 | 0.94 | 0.96 | 0.98 | 0.99 | 0.99 | 1.00 | 1.00 | 1.00 | 1.00 |
|  |  |  |  |  |  |  |  |  |  |  |  |  |  |  |  |  |
|  |  |  |  |  |  |  |  |  |  |  |  |  |  |  |  |  |
| **Eigenvalues** | Observed | 7.76 | 2.38 | 1.32 | 1.04 | 0.71 | 0.49 | 0.43 | 0.27 | 0.22 | 0.17 | 0.11 | 0.04 | 0.03 | 0.02 | 0.01 |
|  | Brocken-stick | 3.32 | 2.32 | 1.82 | 1.48 | 1.23 | 1.03 | 0.87 | 0.73 | 0.60 | 0.49 | 0.39 | 0.30 | 0.22 | 0.14 | 0.07 |
