# Supplementary material for: Contrasting Taxonomic and Phylogenetic Diversity Responses to Forest Modifications: Comparisons of Taxa and Successive Plant Life Stages in South African Scarp Forest
Source: PLoS One. 2015 Feb 26;10(2):e0118722. doi: 10.1371/journal.pone.0118722 (PMC4342016; doi:10.1371/journal.pone.0118722)
Supplement: S3 Table — Shown are effects of forest disturbance, forest loss and spatial trends on phylogenetic α-diversity of species groups. Note the similarity in effect directions and effect sizes to those based on the Rao index shown in Table 1. (DOC) [file pone.0118722.s004.doc]

**Table S3. Using mean pairwise phylogenetic distances (MPD) instead of the Rao index for phylogenetic α-diversity gives similar results.** Shown are effects of forest disturbance, forest loss and spatial trends on phylogenetic α-diversity of species groups. Note the similarity in effect directions and effect sizes to those based on the Rao index shown in Table 1.

|  | **Estimate** | **SE** | **Z** | **p** | **Akaike weight** |
| --- | --- | --- | --- | --- | --- |
|  |  |  |  |  |  |
| **Plants** |  |  |  |  |  |
| Intercept | 0.171 | 0.116 | 1.44 | 0.149 |  |
| **Forest disturbance** | **-0.331** | **0.122** | **2.67** | **0.00766** | **0.69** |
| Forest loss | 0.239 | 0.142 | 1.66 | 0.0979 | 0.48 |
| Easting | 0.666 | 0.509 | 1.30 | 0.193 | 0.59 |
| Northing | -0.622 | 0.663 | 0.936 | 0.350 | 0.46 |
|  | |  |  |  |  |
|  |  |  |  |  |  |
| **Birds** |  |  |  |  |  |
| Intercept | -1.323 | 0.186 | 6.77 | < 0.001 |  |
| Forest loss | 0.241 | 0.232 | 0.987 | 0.324 | 0.31 |
| **Northing** | **-0.462** | **0.217** | **2.03** | **0.0422** | **0.53** |
| **Easting** | **-0.440** | **0.208** | **2.02** | **0.0437** | **0.47** |
