# Supplementary material for: Contrasting Taxonomic and Phylogenetic Diversity Responses to Forest Modifications: Comparisons of Taxa and Successive Plant Life Stages in South African Scarp Forest
Source: PLoS One. 2015 Feb 26;10(2):e0118722. doi: 10.1371/journal.pone.0118722 (PMC4342016; doi:10.1371/journal.pone.0118722)
Supplement: S4 Table — Each repetition was based on one of 1000 phylogenetic trees per species group. The last column gives information on the number of analyses in which a predictor appeared in the final averaged model. Note that predictors that appeared in each analysis (n = 1000) also appear in Table 1b, and that effect sizes are comparable to those based on mean phylogenetic distance matrices of phylogenetic trees from the posterior distributions (Table 1b). Statistically significant predictors (p < 0.050) are shown in boldface type. (DOC) [file pone.0118722.s005.doc]

**Table S4. Means of test statistics from 1000 repetitions of statistical analyses (see Methods) on variation in phylogenetic α-diversity of life stages of adult trees, saplings and seedlings and of birds.** Each repetition was based on one of 1000 phylogenetic trees per species group. The last column gives information on the number of analyses in which a predictor appeared in the final averaged model. Note that predictors that appeared in each analysis (n = 1000) also appear in Table 1b, and that effect sizes are comparable to those based on mean phylogenetic distance matrices of phylogenetic trees from the posterior distributions (Table 1b). Statistically significant predictors (p < 0.050) are shown in boldface type.

|  | **Estimate** | **SE** | **Z** | **p** | **N_included_** |
| --- | --- | --- | --- | --- | --- |
|  |  |  |  |  |  |
| **Plants** |  |  |  |  |  |
| Intercept (= Adult trees) | 2.19 | 0.0552 | 39.1 | < 0.001 | 1000 |
|  |  |  |  |  |  |
| *Successive life stage* |  |  |  |  |  |
| **Saplings** | **-2.84** | **0.0669** | **41.7** | **< 0.001** | **1000** |
| **Seedlings** | **-2.98** | **0.0677** | **43.3** | **< 0.001** | **1000** |
|  |  |  |  |  |  |
| **Forest disturbance** | **-0.193** | **0.0666** | **2.94** | **0.0149** | **1000** |
|  | |  |  |  |  |
| *Successive life stage × Forest disturbance* | |  |  |  |  |
| Saplings × Forest disturbance | -0.098 | 0.0659 | 1.47 | 0.149 | 623 |
| Seedlings × Forest disturbance | -0.132 | 0.0664 | 1.96 | 0.0544 | 623 |
| Forest loss | 0.0861 | 0.0512 | 1.66 | 0.104 | 1000 |
| Easting | -0.0432 | 0.209 | 0.206 | 0.841 | 1000 |
|  |  |  |  |  |  |
| *Successive life stage × Easting* |  |  |  |  |  |
| Saplings × Easting | -0.122 | 0.0670 | 1.80 | 0.0727 | 3 |
| Seedlings × Easting | -0.122 | 0.0670 | 1.80 | 0.0727 | 3 |
|  |  |  |  |  |  |
| Northing | 0.192 | 0.153 | 1.25 | 0.214 | 1000 |
|  |  |  |  |  |  |
| *Successive life stage × Northing* |  |  |  |  |  |
| Saplings × Northing | -0.119 | 0.0661 | 1.77 | 0.0803 | 891 |
| Seedlings × Northing | -0.118 | 0.0662 | 1.76 | 0.0817 | 891 |
|  |  |  |  |  |  |
|  |  |  |  |  |  |
| **Birds** |  |  |  |  |  |
| Intercept | -1.450 | 0.171 | 8.06 | < 0.001 | 1000 |
| Forest loss | 0.330 | 0.211 | 1.49 | 0.139 | 1000 |
| **Easting** | **-0.476** | **0.181** | **2.52** | **0.0126** | **532** |
| **Northing** | **-0.630** | **0.217** | **2.79** | **0.00535** | **1000** |
