# Supplementary material for: Contrasting Taxonomic and Phylogenetic Diversity Responses to Forest Modifications: Comparisons of Taxa and Successive Plant Life Stages in South African Scarp Forest
Source: PLoS One. 2015 Feb 26;10(2):e0118722. doi: 10.1371/journal.pone.0118722 (PMC4342016; doi:10.1371/journal.pone.0118722)
Supplement: S5 Table — Each test was based on one of 1000 phylogenetic trees per species group. Correlations were conditioned on a spatial distance matrix of study plots. Shown are means and standard deviations of Pearson’s correlation coefficient and p-values. Correlations with pmean < 0.050 are shown in boldface type. Note that effect sizes are comparable to those from the analysis based on a mean phylogenetic distance matrix instead of the posterior distribution (Table 2). (DOC) [file pone.0118722.s006.doc]

**Table S5. Results of 1000 partial mantel tests on changes in phylogenetic beta diversity of life stages of plants and of birds.** Each test was based on one of 1000 phylogenetic trees per species group. Correlations were conditioned on a spatial distance matrix of study plots. Shown are means and standard deviations of Pearson’s correlation coefficient and p-values. Correlations with p_mean_ < 0.050 are shown in boldface type. Note that effect sizes are comparable to those from the analysis based on a mean phylogenetic distance matrix instead of the posterior distribution (Table 2).

| **Source of variation** | **Species group** | **r_mean_** | **r_SD_** | **p_mean_** | **p_SD_** |
| --- | --- | --- | --- | --- | --- |
| **Forest disturbance** | Trees | -0.271 | 0.0241 | 0.999 | 0.00106 |
|  | Saplings | 0.105 | 0.0119 | 0.0883 | 0.0235 |
|  | Seedlings | -0.00756 | 0.00714 | 0.492 | 0.0353 |
|  | Birds | 0.0297 | 0.0191 | 0.363 | 0.0690 |
| **Forest loss** | Trees | 0.106 | 0.0224 | 0.107 | 0.0493 |
|  | Saplings | 0.0168 | 0.0108 | 0.403 | 0.0560 |
|  | Seedlings | **0.191** | **0.00592** | **0.0223** | **0.00560** |
|  | Birds | -0.150 | 0.0130 | 0.938 | 0.0181 |
